# CD109 mediates tumorigenicity and cancer aggressiveness via regulation of EGFR and STAT3 signalling in cervical squamous cell carcinoma

**DOI:** 10.1038/s41416-020-0922-7

**Published:** 2020-06-08

**Authors:** Xue-Tang Mo, Thomas Ho-Yin Leung, Hermit Wai-Man Tang, Michelle Kwan-Yee Siu, Peter Kok-Ting Wan, Karen Kar-Loen Chan, Annie Nga-Yin Cheung, Hextan Yuen-Sheung Ngan

**Affiliations:** 1 Department of Obstetrics and Gynaecology, LKS Faculty of Medicine, The University of Hong Kong, Pokfulam, Hong Kong, Special Administrative Region of China; 2 Department of Pathology, LKS Faculty of Medicine, The University of Hong Kong, Pokfulam, Hong Kong, Special Administrative Region of China

**Keywords:** Cervical cancer, Growth factor signalling

## Abstract

**Background:**

CD109 was involved in the tumorigenesis and progression of various cancers via TGF-β1 signalling and STAT3 activation. As CD109 is strongly expressed in cervical squamous cell carcinoma, this study was conducted to investigate its functional characteristics in cervical cancer.

**Methods:**

CD109 expression was examined by immunohistochemistry (IHC) with cervical tissue microarray. The effects of CD109 expression were examined on migration, cell proliferation, spheroid formation and soft-agar colony-formation assay. Meanwhile, cervical cancer cell lines with high CD109 expression were chosen for the functional study using siRNA knockdown and CRISPR/Cas9 knockout.

**Results:**

IHC demonstrated an upregulation of CD109 in the cell membrane of cervical squamous cell carcinoma. CD109( + ) cells isolated by flow-cytometric sorting displayed enhanced migration, cell proliferation, sphere-forming and anchorage-independent cell growth ability. In contrast, silencing of CD109 expression could reverse the in vitro and in vivo tumorigenic and aggressive properties. Furthermore, CD109 induced EGFR-mediated STAT3 phosphorylation known to be responsible for cell migration, proliferation and maintenance of CSC phenotype.

**Conclusion:**

Abundant CD109( + ) populations in cervical cancer cells potentially contributed to carcinogenesis and aggressiveness, whereas silencing of CD109 expression could reverse those properties. CD109 mediates cervical tumorigenicity and aggressiveness via CD109/EGFR/STAT3 signalling.

## Background

Cervical cancer is a common genital tract cancer associated with amounts of cancer-related deaths among women worldwide. Infection with carcinogenic human papillomavirus (HPV) is considered to have an increasing risk of cervical carcinogenesis. Abundant screening and vaccination are beneficial for reducing the disease burden. Based on different stages of carcinoma, its treatment includes surgery, chemotherapy, radiotherapy and immunotherapy, and sometimes in combination. However, for the recurrence and therapeutic resistance of advanced cervical cancer, the molecular mechanisms underlying carcinogenesis and aggressiveness are not fully understood.

CD109 is a glycosylphosphatidylinositol-linked glycoprotein that belongs to the α2-macroglobulin (α2M)/C3, C4 and C5 complement superfamily.^1^ Upregulation of CD109 was found in pancreatic cancer,^2^ hepatocellular carcinoma,^3^ malignant melanoma^4^ as well as in squamous cell carcinomas of the uterine cervix,^5^ lung,^6,7^ oesophagus,^6,8^ stomach,^6^ oral cavity,^9^ gallbladder,^10^ penis^11^ and skin,^12^ which suggested that CD109 might be a cancer-associated marker in various carcinoma models. Furthermore, it has been pointed out that CD109 was associated with the tumorigenicity and carcinogenesis of several human carcinomas.

As a component of the transforming growth factor-β (TGF-β) receptor system, CD109 negatively modulates TGF-β signalling receptor activity in a ligand-specific manner.^13,14^ Meanwhile, CD109 has also been reported to directly interact with epidermal growth factor receptor (EGFR), which would enhance EGF signalling.^15,16^ Moreover, it was hypothesised that CD109 may affect the dimerisation of EGFR, and subsequently induced the Signal Transducers and Activators of Transcription 3 (STAT3) phosphorylation.^16^ CD109 was considered to be involved in the development of human keratinocytes,^17^ glioblastoma cells,^15^ oral cancer^9^ and lung cancer^18^ via regulating the TGF-β1 or EGFR signalling or STAT3 activation. However, the functional role of CD109 in cervical cancer remains unknown.

It had been reported that CD109 mRNA was strongly expressed in cervical squamous cell carcinoma by qPCR.^5^ For this reason, we accordingly used C33A, C4-1, CaSki and SiHa, which are squamous cell carcinoma cell lines of uterine cervix to investigate the functional role of CD109 in cervical cancer. Firstly, we found that high expression level of CD109 protein was also observed in cervical squamous cancer tissues in IHC analysis. Then, we characterised the functional role of CD109 in cervical squamous carcinoma cell lines, and evaluated the effects on the blockade of CD109 via in vitro and in vivo functional assays. Our findings indicated that CD109 facilitates tumorigenicity and cancer aggressiveness, which might be mediated by EGFR/STAT3 signalling in cervical squamous cell carcinoma.

## Methods

### Cell culture

Four cervical squamous carcinoma cell lines, C33A, C4-1, CaSki and SiHa, were obtained from the American Type Cell Collection (ATCC, Manassas, VA) and cultured in modified Eagle’s medium (Gibco-BRL, Gaithersburg, MD) with 10% foetal bovine serum (Gibco) and 1% penicillin–streptomycin (Gibco). All cell lines were incubated at 37 °C and 5% CO_2_ in a humidified atmosphere.

### RNA interference

C4-1, Caski or SiHa cells were seeded into a six-well plate at the density of 6 × 10^5^ cells/well, and were transfected with either non-target control (NTC) siRNA or CD109 siRNA (1 μM each, ID#s43925, Silencer^®^ Select, Life Technologies Corp.) using Lipofectamine RNAiMAX reagent (Life Technologies) according to the manufacturer’s instructions. After 72 h of incubation, cells were subjected to western blotting and in vitro functional assays.

### Generation of CD109–CRISPR-knockout lines

Using online design tools, including Zhang Lab, MIT 2015 (http://crispr.mit.edu/), *CRISPOR* (http://crispor.tefor.net/, Version 4.3), *CHOPCHOP* (https://chopchop.rc.fas.harvard.edu) and Thermo Fisher (Thermo Fisher Scientific, Waltham, MA, USA) GeneArt^TM^ (https://apps.thermofisher.com/crispr/index.html?icid=fr-crispr-3#/search), we designed the CRISPR target for generating knockout cell lines. CRISPR guide RNAs (gRNAs) targeting an early coding exon of CD109 were selected. Three gRNAs were chosen for the genomic knockout of CD109. For CRISPR/Cas9 gene editing, CaSki and SiHa cells highly expressing the CD109 protein were co-transfected with gRNA (GeneArt^®^CRISPR U6 Strings™ DNA gRNA, Life Technologies) and *Cas9* protein (GeneArt™ Platinum™ Cas9 Nuclease) using Lipofectamine CRISPRMAX reagent. After 72-h transfection, the cutting of Cas9 at the CD109 gene region and the cleavage efficiency of each gRNA were examined by a genomic cleavage detection assay using a GeneArt^®^ Genomic Cleavage Detection Kit (Life Technologies). The transfected cells with the highest cleavage efficiency were selected for the isolation of CD109-KO clones. Single-cell isolation was achieved by seeding the transfected cells into 96-well plates via serial dilution. For secondary screening of knockout clones, a genomic cleavage detection assay was performed to select knockout clones. The pGEM^®^-T Easy vector system (Promega) was used for cloning PCR products amplified from each isolated clone. Sequencing of the edited target site of the CD109 gene was performed to determine the frameshifting mutation in the isolated clones. Furthermore, the off-targets sites were predicted using *CRISPOR* (http://crispor.tefor.net/) and *Benchling* (https://benchling.com/crispr). Sequencing of the potential off-targets was also performed in the isolated clones.

### Western blotting

Proteins were determined by western blotting using primary antibodies for CD109 (1:200, C-9, sc-271085, Santa Cruz Biotechnology, Inc., Santa Cruz, CA), EGFR (C74B9, 1:2000, no. # 2646, Cell Signalling Technology), STAT3 (1:1000, no. #9132, Cell Signalling Technology), pSTAT3 (Ser727, 1:1000, no. #9134, Cell Signalling Technology), Bmi1 (D20B7, 1:1000 no. #6964, Cell Signalling Technology), Nanog (D73G4, 1:1000, no. #4903, Cell Signalling Technology), Oct-4A (C30A3, 1:1000, no. #2840, Cell Signalling Technology) and a horseradish peroxidase-conjugated secondary antibody. The binding signal was then visualised by enhanced chemiluminescence (ECL). β-actin (1:6000, # MA5-15739, Thermo Fisher Scientific, Waltham, MA, USA) was used as an internal loading control.

### Immunohistochemical analysis of tissue microarray

The CXC1501 and CXC1502 arrays (Pantomics Inc., Richmond, CA, USA) consist of 300 cores from normal–benign (10 cases) and cancer (140 cases) tissues in duplicate. For immunohistochemical (IHC) analysis, formalin-fixed paraffin-embedded cervical cancer tissue arrays (CXC1501 and CXC1502) were immunostained with primary monoclonal anti-CD109 (C-9, sc-271085, Santa Cruz Biotechnology, Inc.) in 1:100 dilution. DAKO EnVision^TM^ system (Carpentaria, CA, USA) was used for immunodetection. The slides were counterstained with haematoxylin and scanned by Aperio Scanscope (Leica Biosystems, Vista, CA, USA). The tissue microarray was analysed individually. The expression of CD109 was determined by intensity and abundance (positivity) using Image Scope with a pixel count algorithm.

### Flow cytometry

Flow-cytometric analysis was performed to examine the percentage of CD109( + ) cells in four cervical squamous carcinoma cell lines, C33A, C4-1, CaSki and SiHa. To isolate CD109( + ) and CD109(–) populations, cells were labelled with PE-conjugated CD109 antibody (*#* 12-1099-42, eBioscience, Thermo Fisher Scientific, Waltham, MA). Isotype-matched mouse immunoglobulin was used as control. Cells were analysed and isolated via fluorescence-activated cell sorting (FACS) Aria Flow cytometer (BD Bioscience, San Jose, CA). The purity of sorted CD109( + ) and CD109(–) cells was evaluated by flow-cytometric analysis. Data were analysed by FlowJo v10.4 (TreeStar, Palo Alto, CA).

### XTT cell proliferation assay

To determine cell viability, cervical cancer cells were seeded in a 96-well plate on day 0. The rate of cell growth was determined by XTT Cell proliferation kit (Roche) for 4 days according to the manufacturer’s protocol. The cell proliferation rate was determined and compared with the control cells.

### Soft-agar colony-formation assay

A soft-agar assay was performed to investigate anchorage-independent growth of the cervical cancer cells. A total of 2500 cells were suspended in 2 ml of medium (MEM supplemented with 10% FBS) containing 0.6% agar. The cell mixture was then seeded, three wells for each condition, onto a layer of 2% bottom agar in a six-well plate. After 4 weeks of incubation, colonies were counted in four fields for each well.

### Transwell cell migration assay

To estimate cell migration ability using transwell migration assay, a cell suspension containing 1.0 × 10^5^ cells in 100 µl of serum-free medium was seeded into the top chamber (Corning, NY, USA), while 600 µl of complete medium was added as a chemoattractant to the bottom chamber. The 24-well plates were incubated for 24–28 h at 37 °C at 5% CO_2_. Cells that migrated through the pores in the membrane were fixed and stained with crystal violet. Three independent sections in each transwell filter were visualised under the microscope (TE300, Nikon), and the number of migrated cells was counted.

### Spheroid-formation assay

The spheroid-formation assay was performed to evaluate the effect of CD109 expression on sphere-forming ability in cervical cancer cells. Cervical cancer cells were seeded at 2500 cells per well in six-well ultra-low-attachment plates (Costar) and cultured in Dulbecco’s Modified Eagle Medium: Nutrient Mixture F-12 (DMEM/F-12, Gibco) containing 0.4% BSA (Sigma), 5 μg/ml insulin (Sigma), 20 ng/ml human recombinant EGF (Biosource) and 10 ng/ml bFGF (Biosource). After 7–10 days, the spheroids were visualised under the microscope (TE300, Nikon), and their numbers and sizes were recorded and compared with control cells.

### Tumour xenograft mouse model

To investigate whether CD109 knockout (KO) reduces cervical tumorgenicity ability in vivo, CaSki control cells and CD109-KO clone (Q4), SiHa control cells and CD109-KO clone (L65) cells at a concentration of 1 × 10^6^ cells/100 μl were injected subcutaneously into the flanks of female BALB/c nude mice (4–6 weeks, five mice per group). After injection, the mice were housed in cages under specific pathogen-free conditions with continuous supply of sterile air at 25 °C by Laboratory Animal Unit (LAU) of The University of Hong Kong. The housing condition and health status of the nude mice were regularly monitored by LAU staff. The size of tumour formed (diameters) was monitored and measured for every 2–3 days since day 7. Six weeks after the injection, mice were sacrificed with 80 mg/kg pentobarbital euthanised and dissected for tumour collection. The animal experiments were conducted according to the guidelines approved by the University of Hong Kong Committee on the Use of Live Animals in Teaching and Research (CULATR No. 3921-16).

### Data analysis

Graphs and statistics were generated using the GraphPad Prism software (GraphPad Software Inc., San Diego, CA, USA). Student’s *t* test (for parametric data) and Mann–Whitney test (for non-parametric data) were applied for comparing data between two groups. Kruskal–Wallis rank test was performed for multiple non-parametric data comparisons. A *P* value<0.05 was considered statistically significant.

## Results

### Cervical squamous cell carcinoma showed high expression of CD109 by IHC analysis of TMAs

**Fig. 1:**
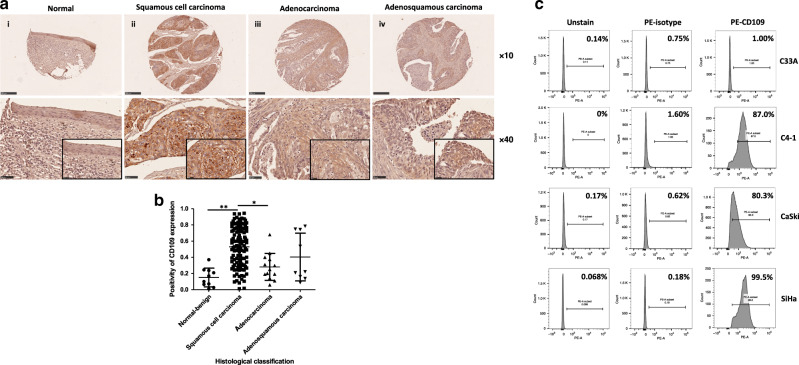
Upregulation of CD109 was frequently detected in cervical squamous cell carcinoma. **a** Representative immunohistochemical CD109 staining of cervical cancer tissue microarray sections: weak CD109 staining in normal cervical tissue (i), strong CD109 staining in squamous cell carcinoma (ii), mild or moderate CD109 staining in adenocarcinoma (iii) and adenosquamous cell carcinoma (iv). Magnification: upper, ×10; lower, ×40. The insets highlight regions with ×80 magnification. **b** Dot plot showing the distribution of CD109 immunoreactivity (presented as positivity, mean ± SD) in different histological classification groups. ^**^P_1_ < 0.0001, ^*^P_2_ = 0.0007, Mann–Whitney test. **c** CD109( + ) subpopulation in cervical cancer cell lines C33A, C4-1, CaSki and SiHa was examined by flow cytometry.

By IHC analysis of two cervical tissue microarrays (CXC1501 and CXC1502), we determined the expression level of CD109 protein in 10 normal–benign cervical tissue samples and 140 cervical carcinoma tissue samples. Moderate-to-strong CD109 staining was observed at the cellular membrane within cervical squamous cancer cells, while weak CD109 staining was displayed in normal–benign cervical tissue (Fig. 1a). CD109 immunoreactivity was significantly different among various histological classifications (*P* < 0.0001, Supplementary Table S1). In particular, CD109 immunoreactivity was significantly higher in the squamous cell carcinoma cases than in the normal–benign cervical tissue cases (*P*_1_ < 0.0001, Mann–Whitney test) and adenocarcinoma cases (*P*_2_ = 0.0007, Mann–Whitney test) (Fig. 1b). However, no significant difference of CD109 expression was found among different clinical TNM stages/grade groups. Our findings suggested that CD109 protein was frequently overexpressed in cervical squamous cell carcinoma.

### Flow-cytometric analysis of the CD109(+) subpopulation in various cervical cancer cell lines

The subpopulation of cells expressing CD109 was examined by flow-cytometric analysis in multiple cervical squamous carcinoma cell lines (C33A, C4-1, CaSki and SiHa). The percentages of CD109( + ) cells in each line were determined. The CD109( + ) subpopulation was high in C4-1 (87.0%), CaSki (80.3%) and SiHa (99.5%) cell lines. In contrast, in the C33A cell line, only 1.0% CD109( + ) population was detected (Fig. 1c).

### Isolation and functional characterisation of the sorted CD109(+) and CD109(–) cells from C4-1 and CaSki lines

**Fig. 2:**
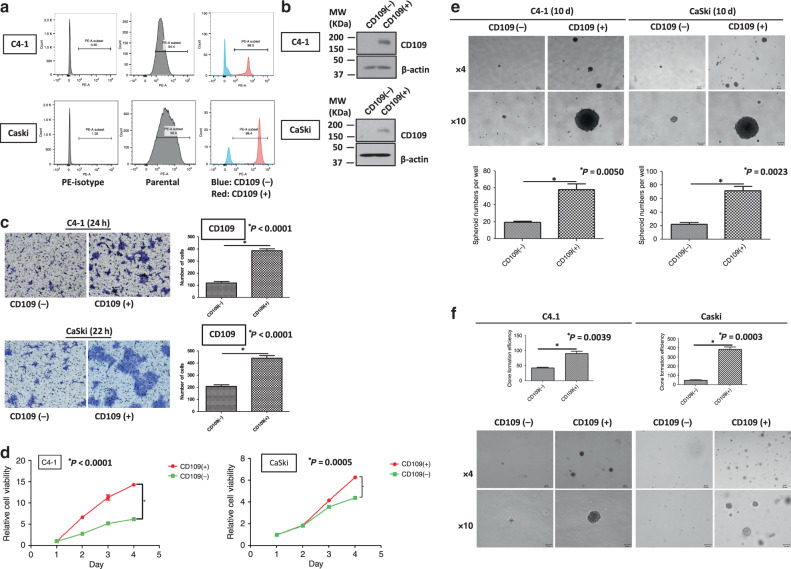
Sorted CD109(+) cells displayed enhanced carcinogenic and aggressive abilities. The purity of CD109- expressed cells in sorted CD109(−) and CD109( + ) subpopulations was confirmed by flow-cytometric (**a**) and western blot analysis (**b**). **c** A representative image (left) and quantification bar graph (right) of migrated sorted CD109(−) and CD109( + ) subpopulations in C4-1 and CaSki cells (crystal violet). **d** Proliferation analyses of sorted CD109(−) and CD109( + ) subpopulations in C4-1 and CaSki cells. ^*^P_*C4-1*_ < 0.0001, *P*_*CaSki*_ = 0.0005, using the Student’s *t* test. **e** Representative images of spheroids formed by sorted CD109(−) and CD109( + ) cells (above, magnification, ×4, ×10). Bar graph (below) represents the mean number of spheroids from each well under the microscope, and error bars represent SD, ^*^P_*C4-1*_ = 0^.^005, *P*_*CaSki*_ = 0.0023. **f** CD109- enhanced anchorage-independent cell growth ability was confirmed by soft-agar colony-formation assay. The sorted CD109( + ) cells formed larger and more clones than CD109(−) cells. ^*^P_*C4-1*_ = 0^.^0039, *P*_*CaSki*_ = 0.0003, using the Student’s *t* test.

CD109( + ) and CD109(–) cells were isolated from C4-1 and CaSki cell lines using PE-conjugated CD109 antibody by sorting on a BD FACSAria flow cytometer (BD Bioscience). In C4-1 cells, the purity of CD109-expressing cells was 96.0% in the sorted CD109( + ) subpopulation, while only 2.85% cells with CD109 expression in the sorted CD109(−) subpopulation. It was also confirmed that 98.4% of the sorted CD109( + ) cells expressed CD109, whereas 8.16% of the sorted CD109(−) cells expressed CD109 in CaSki cells (Fig. 2a). The expression level of CD109 in the sorted CD109( + ) and CD109(–) cells was further validated by western blotting (Fig. 2b).

Next, a cell migration assay was performed and revealed that the sorted CD109( + ) cells had remarkably enhanced migration ability (Fig. 2c, *P*_*C4-1*_ < 0.0001, *P*_*CaSki*_ < 0.0001). Furthermore, the XTT cell proliferation assay showed that the sorted CD109( + ) cells grew dramatically faster than the CD109(–) cells, in both C4-1 and CaSki lines (Fig. 2d, *P*_*C4-1*_ < 0.0001, *P*_*CaSki*_ = 0.0005). Spheroid-formation assay indicated that CD109( + ) cells had the ability to form more and larger spheroids than the CD109(–) cells (Fig. 2e, *P*_*C4-1*_ = 0.005, *P*_*CaSki*_ = 0.0023). Enhanced anchorage-independent cell growth capability was observed in the CD109( + ) cells, which formed more and larger colonies within 6–8 weeks (Fig. 2f, *P*_*C4-1*_ = 0.0039, *P*_*CaSki*_ = 0.0003). These results indicated that CD109( + ) populations demonstrated an enhanced cell migration, proliferation, sphere-forming and anchorage-independent cell growth ability.

### Functional characterisation of cervical cancer cells with CD109 siRNA knockdown

**Fig. 3:**
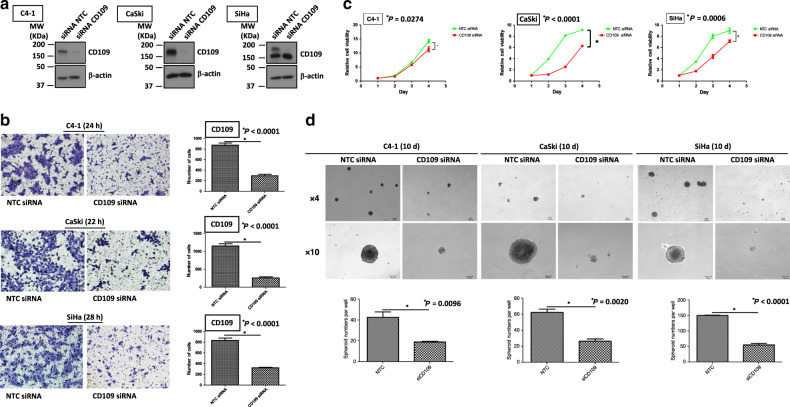
siRNA-mediated CD109 knockdown suppressed migration, cell proliferation and sphere-forming ability. **a** Western blotting for confirmation of CD109 siRNA knockdown in C4-1, CaSki and SiHa cells. **b** A representative image (left) and quantification bar graph (right) of migrated NTC siRNA or CD109 siRNA- knockdown cells (crystal violet). ^*^P_*C4-1*_ < 0.0001, *P*_*CaSki*_ < 0.0001, *P*_*SiHa*_ < 0.0001, using the Student’s *t* test. **c** Proliferation analyses of NTC siRNA or CD109 siRNA-knockdown cells. ^*^P_*C4-1*_ = 0.0274, *P*_*CaSki*_ < 0.0001, *P*_*SiHa*_ = 0.0006. **d** Representative images of spheroids formed by NTC siRNA or CD109 siRNA-knockdown cells (above, magnification, ×4, ×10). Bar graph (below) represents the mean number of spheroids from each well under the microscope, and error bars represent SD, ^*^P_*C4-1*_ = 0.0096, *P*_*CaSki*_ = 0.0020, *P*_*SiHa*_ < 0.0001.

C4-1, CaSki and SiHa cells with high CD109 expression were chosen for functional study using a loss-of-gene function approach through siRNA knockdown to determine whether CD109 is physiologically relevant in cervical cancer. The knockdown efficiency of CD109 was examined by western blotting. It has been reported that various types of CD109 could be detected in the cells, which included the uncleaved cytosolic immature CD109 (190 kDa), cleaved soluble form (180 kDa) and membrane-attached form (25 kDa).^19,20^ A reduction of CD109 protein (the upper 190-kDa band) has been found in these three cell lines (Fig. 3a). Interestingly, we found that the lower 180-kDa band was not decreased in SiHa cells. Zhou et al.^21^ have also reported a similar pattern of CD109 expression in different percentages of CD109-sorted squamous carcinoma A431 cells. Based on their findings, the lower band of CD109 protein did not change in 10–90% sorted CD109 A431 cells, whereas it decreased in lower 10% cells. The reason for such observation in some cell lines needs to be determined in future studies. The transwell cell migration assay revealed that CD109 knockdown suppressed cell migration (Fig. 3b, *P*_*C4-1*_ < 0.0001, *P*_*CaSki*_ < 0.0001, *P*_*SiHa*_ < 0.0001). Furthermore, CD109-knockdown cells exhibited slower cell proliferation (Fig. 3c, *P*_*C4-1*_ = 0.0274, *P*_*CaSki*_ < 0.0001, *P*_*SiHa*_ = 0.0006), as demonstrated by XTT assay. Suppression in self-renewal ability was observed in the CD109-knockdown cells, which formed smaller and fewer spheroids in all three cell lines (Fig. 3d, *P*_*C4-1*_ = 0.0096, *P*_*CaSki*_ = 0.0020, *P*_*SiHa*_ < 0.0001). These results suggested that knockdown of CD109 suppressed cell migration, cell proliferation and self-renewal ability in cervical squamous carcinoma cells.

### Establishment of CD109-knockout (KO) clones in CaSki and SiHa cells

**Fig. 4:**
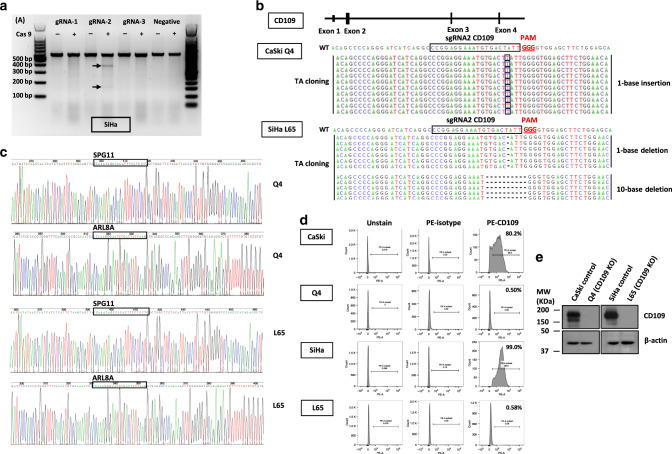
Generation of CD109-CRISPR/Cas9 knockout clones. **a** The efficiency of the targeted deletion with CRISPR/CAS9 was determined by PCR in SiHa cells. Black arrows denote the cleaved band intensity of the transfected cells. **b** A schematic diagram depicting the CD109-guide RNA (gRNA2)-targeting region at exon 2 of CD109 (NC_000006.12) on chromosome 6; the PAM sequence is shown in red. The efficiency and precision of targeted knockout in CD109 were confirmed by TA-cloning sequencing analyses. TA-cloning DNA sequences of the wild-type (WT) and mutant sequences in KO clones were named Q4 and L65. Representative sequences for TA cloning are shown. Red boxes highlighted 1-base-pair (bp) insertion; the short black dash denotes 1-bp or 10-bp deletions. **c** Sequencing results of the predicted off‐target genes (SPG11 and ARL8A) in Q4 and L65 cells. **d** The CD109 ( + ) subpopulation percentage in CD109 CRISPR/Cas9-knockout clones Q4 and L65 was examined by flow cytometry. **e** Western blot analysis for confirmation of CD109 CRISPR/Cas9 knockout in CaSki and SiHa cells.

To further confirm whether the carcinogenic and aggressive properties can be reversed by eliminating CD109 expression, CaSki and SiHa cell lines were chosen for functional study using CRISPR/Cas9 knockout. Three gRNAs were designed for CD109. Each of the gRNAs and Cas9 protein was introduced into CaSki and SiHa cells via transfection. As shown in Fig. 4a, the cleaved band intensity of the transfected cells indicated that the highest cleavage efficiency was found in the gRNA2-transfected SiHa cells. Therefore, CD109–gRNA2/Cas9-transfected cells were chosen for the establishment of CD109-KO clones in CaSki and SiHa cells. CD109-KO clone named Q4 was selected from screening of 100 isolated clones in CaSki cells, and CD109-KO clone L65 was selected from SiHa. The efficiency and precision of targeted knockout in CD109 were further confirmed by TA cloning and sequencing analyses. The sequencing results showed 1-base-pair (bp) insertion at exon 2 of CD109 in the Q4 cells, and 1-bp or 10-bp deletions of that in the L65 cells (Fig. 4b). To determine whether mutagenesis occurred at the potential off-target sites, we performed sequencing on 2 predicted off-target genes *SPG11* and *ARL8A*, which have high homology to the CD109–gRNA2/Cas9 target site. No mutation was observed in these genes in the Q4 and L65 KO clones (Fig. 4c). To confirm the elimination of CD109 expression by CRISPR/Cas9 KO, the percentage of CD109( + ) subpopulation was examined by flow cytometry. We found that the percentages of CD109( + ) subpopulation in Q4 and L65 clones were 0.50% and 0.58%, respectively (Fig. 4d). The knockout efficiency was also confirmed by western blotting, and no endogenous CD109 expression was found in Q4 and L65 cells (Fig. 4e). CD109 KO was deemed successfully in the Q4 and L65 cells.

### Functional characterisation of CD109-knockout clones Q4 and L65

**Fig. 5:**
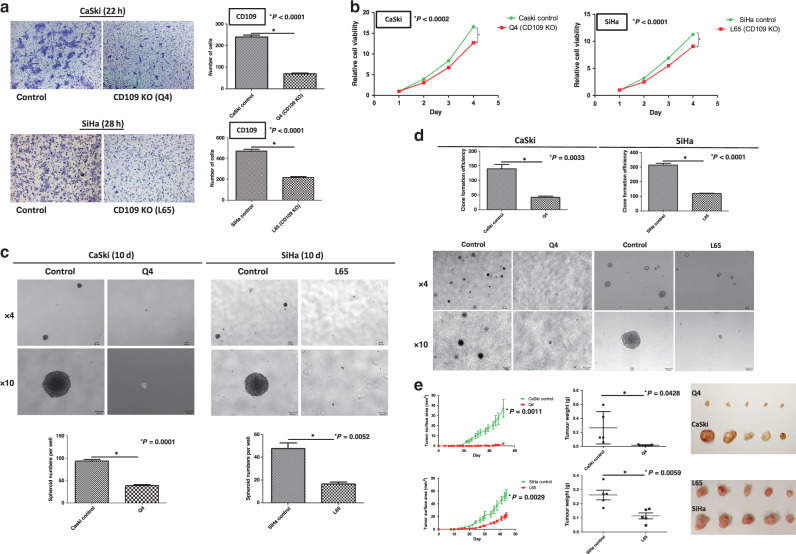
CRISPR knockout of CD109 reversed the in vitro and in vivo tumorigenic and aggressive properties. **a** A representative image (left) and quantification bar graph (right) of migrated CaSki control or Q4-CD109-KO cells, SiHa control or L65-CD109-KO cells (crystal violet). ^*^P_*CaSki*_ < 0.0001, *P*_*SiHa*_ < 0.0001. **b** Proliferation analyses of CaSki control or Q4, SiHa control or L65 cells. ^*^P_*CaSki*_ = 0.0002 and *P*_*SiHa*_ < 0.0001, using the Student’s *t* test. **c** Representative images of spheroids formed by CaSki control or Q4, SiHa control or L65 cells (above, magnification, ×4, ×10). Bar graph (below) represents the mean number of spheroids from each well under the microscope, and error bars represent SD, ^*^P_*CaSki*_ = 0.0001, *P*_*SiHa*_ = 0.0052. **d** The KO of CD109 suppressed tumorigenicity in vitro, as shown by a soft-agar colony-formation assay. CD109-KO (Q4 and L65) cells formed smaller and fewer clones than CaSki and SiHa control cells on soft-agar plate. ^*^P_*CaSki*_ = 0.0033, *P*_*SiHa*_ < 0.0001. **e** The KO of CD109 suppressed tumorigenicity in vivo as demonstrated by nude mouse xenograft assays. The tumours formed by Q4 or L65-CD109-KO cells were smaller (tumour surface area, ^*^P_*CaSki*_ = 0.0011, *P*_*SiHa*_ = 0.0029) and lighter (tumour weight, ^*^P_*CaSki*_ = 0.0428, *P*_*SiHa*_ = 0.0059) than those formed by CaSki or SiHa control cells.

To further investigate the effects on the abrogation of CD109 expression, functional assays were performed in CaSki control cells and Q4-CD109-KO clone, SiHa control cells and L65-CD109-KO clone. The transwell cell migration assay revealed that CD109 KO greatly reduced the number of migrated cells in Q4 (*P* < 0.0001) and L65 (*P* < 0.0001) clones (Fig. 5a). The XTT assay demonstrated that CD109 KO attenuated the cell proliferation rate of Q4 (*P* = 0.0002) and L65 (*P* < 0.0001) cells (Fig. 5b). A spheroid-formation assay was performed to evaluate the self-renewal ability of CD109-KO cells. CD109 knockout almost completely abrogated the capability in forming spheroids: Q4 (*P* = 0.0001) and L65 (*P* = 0.0052) cells (Fig. 5c). Furthermore, suppressed in vitro tumorigenicity was observed in the CD109-KO (Q4 and L65 clone) cells, which formed fewer and smaller colonies within 6 weeks, as shown by a soft-agar assay (*P*_*CaSki*_ = 0.0033, *P*_*SiHa*_ < 0.0001, Fig. 5d). To investigate whether CD109 KO suppressed in vivo tumorigenicity, CaSki control and Q4-CD109-KO cells, SiHa control and L65-CD109-KO cells were subcutaneously injected into nude mice (*n* = 5). The tumours formed by the Q4 and L65 cells were significantly smaller (*P*_*CaSki*_ = 0.0011, *P*_*SiHa*_ = 0.0029) and lighter (*P*_*CaSki*_ = 0.0428, *P*_*SiHa*_ = 0.0059) than tumours formed by the CaSki and SiHa control cells (Fig. 5e). Taken together, these results implied that CRISPR knockout of CD109 resulted in the suppression of cell migration, proliferation and self-renewal ability, and attenuated in vitro and in vivo tumorigenicity.

### CD109-induced EGFR-mediated STAT3 phosphorylation may respond for cell migration, cell growth ability and CSC phenotype maintenance in cervical squamous carcinoma cell lines

**Fig. 6:**
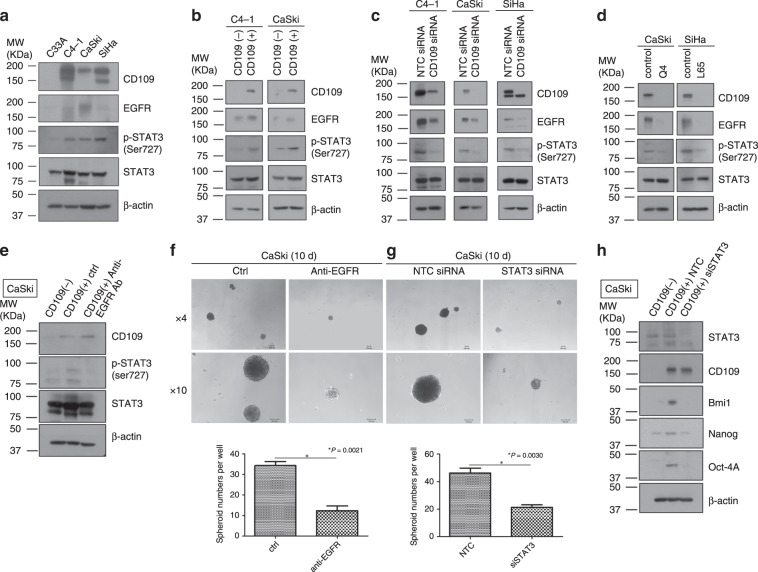
CD109/EGFR/STAT3 signalling may response for cell migration, proliferation and maintenance of CSC phenotype. Western blotting for EGFR expression and phosphorylation of STAT3 on a panel of human cervical cancer cell lines (**a**), sorted CD109(−) and CD109( + ) cells in C4-1 and CaSki lines (**b**), NTC siRNA or CD109 siRNA-knockdown cells in C4-1, CaSki and SiHa lines (**c**) and CaSki control or Q4-CD109-KO cells, SiHa control or L65-CD109-KO cells (**d**). Western blotting for phosphorylation of STAT3 on sorted CD109(−) and CD109( + ) cells with/without anti-EGFR treatment (**e**). Representative images of spheroids formed by sorted CD109( + ) CaSki cells treated w/o EGFR (magnification, ×4, ×10). The bar graph (below) represents the mean number of spheroids from each well under the microscope, and error bars represent SD, ^*^P = 0.0021 (**f**). Representative images of spheroids formed by NTC siRNA or STAT siRNA-knockdown CaSki CD109( + ) cells (magnification, ×4, ×10). The bar graph represents ^*^P = 0.0030 (**g**). Western blotting for stem cell markers on sorted CD109(−), NTC siRNA or STAT siRNA-knockdown CD109( + ) CaSki cells (**h**).

A recent review^16^ pointed out that CD109 affects the activation of signal transducer and activator of transcription factor 3 (STAT3), which might be mediated by epidermal growth factor receptor (EGFR). To investigate whether CD109 facilitates the EGFR/STAT3 signalling, we performed a Western blot analysis to determine the expression of CD109 and EGFR, and the phosphorylation of STAT3 in different cervical cancer cell lines. We found that the cervical squamous carcinoma cell lines that highly expressed CD109 (C4-1, CaSki and SiHa cells), as compared with low CD109-expressing C33A cells, had higher expression levels of EGFR and higher STAT3 phosphorylation (Fig. 6a). Moreover, higher EGFR expression and STAT3 phosphorylation were also detected in the sorted CD109( + ) population over the sorted CD109(–) population (Fig. 6b). Furthermore, suppression of CD109 with siRNA dramatically reduced STAT3 phosphorylation and EGFR expression (Fig. 6c). This regulation of EGFR–STAT3 activity was also observed in CD109-KO clones (Q4 and L65) of CaSki and SiHa cells (Fig. 6d). Taken together, these data suggested that CD109 may be involved in the induction of EGFR-mediated STAT3 phosphorylation.

To further determine the effects of EGFR in mediating CD109/STAT3 activity, the sorted CD109( + ) CaSki cells were treated with anti-EGFR antibody (2 µl/ml, Cetuximab, Cat. #A1047, BioVision) followed by in vitro functional assays. The western blotting demonstrated that the phosphorylation of STAT3 was attenuated in EGFR-blocked CD109( + ) cells (Fig. 6e). The results of the following functional assays indicated that the blockage of EGFR remarkably inhibited cell migration (migration assay, *P* < 0.0001) (Supplementary Fig. S1A), cell growth (XTT assay, *P* < 0.0001) (Supplementary Fig. S1B) and self-renewal ability (spheroid-formation assay, *P* = 0.0021) (Fig. 6f). Then, we performed siRNA knockdown of STAT3 expression in sorted CD109( + ) cells to determine whether suppression of STAT3 recapitulated the phenotypes resulting from knockdown of CD109 expression. Our finding indicated that STAT3 knockdown elicited similar cellular phenotypes as CD109 knockdown, remarkably reducing cell migration (migration assay, *P* = 0.0016) (Supplementary Fig. S1C), cell viability (XTT assay, *P* = 0.0343) (Supplementary Fig. S1D) and self-renewal ability (spheroid-formation assay, *P* = 0.0030) (Fig. 6g). Moreover, enhanced protein expression levels of ‘stemness’ genes, Bmi1, Nanog and Oct-4A, were detected in the sorted CD109( + ) subpopulation, as compared with CD109(−) subpopulation. Besides, knockdown of STAT3 attenuated ‘stemness’ genes’ expression in the CD109( + ) cells (Fig. 6h). STAT3 would be essential for maintaining the expression of those putative stem cell genes. Those results suggested that cell migrative and proliferative functions, and CSC phenotype maintenance of cervical squamous carcinoma cell lines, were regulated via CD109/EGFR/STAT3 signalling.

## Discussion

CD109 is expressed in a subset of haematopoietic cells, bone marrow CD34^+^ cells, platelets, endothelial, activated T cells and mesenchymal stem cells.^16,22^ However, it is rarely expressed in normal human tissue, with the exception of bronchial epithelial, mammary myoepithelial cells, lacrimal and salivary glands and prostate basal cells.^7,23^ As a cancer-associated marker, CD109 was upregulated in various tumour tissues, cancer cell lines and CSCs. Several studies have highlighted CD109 as a potential therapeutic target for cancers, especially for the CSC population of the tumour tissue.^24–28^ A recent study demonstrated strong expression of CD109 in non-invasive urothelial basal cell carcinomas, suggesting that CD109 may be involved in bladder tumorigenesis, and serves as a potential CSC marker in urothelial carcinoma.^26^ Another study documented that high expression of CD109 in ALDH^high^ epithelioid sarcoma cells was critical to maintain tumorigenesis and regulate the phenotype of CSCs.^25^ Furthermore, CD109 + breast CSCs exhibit stronger tumorigenicity compared with CD109– CSCs, which may be involved in progression and metastasis of triple-negative breast cancer.^24^ Jia et al.^27^ also reported that CD109 is expressed in both nasopharyngeal carcinoma (NPC) cells and NPC CSCs, which suggests that targeting CD109 could yield better clinical treatment of the cancer.

For cervical squamous cell carcinomas, as high expression level of the CD109 gene can also be detected, CD109 would be considered as a potential molecular target for the development of new therapies. In cervical cancer, CSC population was found in both human cell lines and primary tumour tissue, and demonstrated a high degree of radioresistance.^29,30^ Consequently, specific detection and targeting of CSCs could be a critical approach in therapy against cervical cancer.^30,31^ Currently, multiple putative stem cell markers, such as ALDH1, CD44, cytokeratin 17 (CK17), C-MYC, Nanog, OCT3/4, Sox2 and STAT3, are considered as candidates for the induction of cervical carcinogenesis.^32^

In our previous study,^33^ we established attached and spheroid cells that were derived from primary cervical tumour tissues. Enhanced expression of multiple ‘stemness’ genes (*Bmi1, Nanog* and *Oct-4*) was detected in spheroid cells as compared with attached cells, which indicated that primary cervical cancer tissue harboured a population of CSCs. Gene expression profiling was performed on the established attached and spheroid cells. We reported higher levels of CD55 and CD109 expression in the spheroid cells than in their attached counterparts.^33^ Based on those findings, we hypothesised that CD109 might serve as a potential marker of CSCs. Therefore, we further investigate the functional role of CD109-expressing cells and their tumorigenic characteristics in cervical cancer.

The qPCR result of a small-sample study (10 cases) showed that the expression level of CD109 mRNA was higher in cervical squamous cell carcinoma than normal tissue and endometrial adenocarcinoma.^5^ We here present the first report of a large-sample IHC analysis of two cervical TMAs, which demonstrated the expression of CD109 in cervical tissues at protein level. Our finding suggested that the expression level of CD109 protein was significantly different among various histological classifications of cervical cancer. CD109 protein was more frequently expressed in cervical squamous cell carcinoma than other histological types of carcinoma. Moreover, CD109 protein expression was enhanced at the cell membrane of a subset of cervical tumour cells. It has been reported that similar pattern of CD109 expression was observed in urothelial carcinoma, in which a strong staining of CD109 expression was found in the basal layer of non-invasive urothelial carcinoma.^26^ For other correlations between clinicopathological factors and CD109 immunostaining, CD109 expression has been pointed out to be associated with the tumour grade in squamous cell carcinoma of vulvar,^34^ epithelioid sarcoma,^25^ triple-negative breast cancer^24^ and urothelial carcinoma.^26^ In addition, significant correlation between CD109 expression and stage could be found in myxofibrosarcoma,^28^ squamous cell carcinoma of the tongue^35^ and hepatocellular carcinoma.^36^ In this study, no significant difference has been found between CD109 expression and different subgroups of clinical TNM stages/grades in cervical cancer.

CD109 was revealed as cell-surface antigen and frequently detected by IHC studies and western blotting using anti-CD109 antibody. In our study, we performed western blotting to determine the expression of CD109 at protein level in different cervical squamous carcinoma cell lines. We found that non-single bands of CD109 protein would be detected in some cervical squamous carcinoma cell lines. Based on previous studies,^19,20^ the upper band was referred to an uncleaved immature form, whereas the lower band was the cleaved form.

Recent studies have also revealed that CD109-positive cells isolated by flow cytometry had higher sphere-forming ability and maintained the similar features of cancer-initiating cell population in epithelioid sarcoma.^25^ Tao et al.^24^ also pointed out that CD109 + CSCs have stronger tumorigenicity, both in vitro and in vivo in triple-negative breast cancer. In our study, we performed flow cytometry to analyse the percentage of CD109( + ) subpopulation in cervical squamous carcinoma cell lines. Then, we examined the effects of CD109 expression status in those cell lines by cell viability, cell invasion, spheroid-formation and soft-agar assay. CD109( + ) population isolated from C4-1 and CaSki cells has enhanced cell migration, proliferation, self-renewal ability and tumorigenicity. These findings indicate that the CD109( + ) population exhibited tumorigenic properties in cervical squamous cell carcinoma.

To examine the physiological relevance of CD109 with cervical squamous cell carcinoma, we knocked down CD109 expression in C4-1, CaSki and SiHa cells using siRNA. Recent studies pointed out that downregulation of CD109 with siRNA inhibited cell growth in squamous cell carcinoma (SCC) of the oral cavity,^9^ and CD109-targeted shRNA knockdown suppressed cell proliferation and induced apoptosis in hepatocellular carcinoma.^3^ Meanwhile, in our study, we observed that transient knockdown of CD109 suppressed the cell migration, proliferation and self-renewal ability. These results suggested that CD109 expression might be associated with the tumorigenesis, progression and metastasis properties of cervical squamous carcinoma cells.

In addition, CD109 knockout using a CRISPR/Cas9 system was performed to further confirm whether the oncogenic properties can be reversed by eliminating CD109 expression in CaSki and SiHa cells. Other studies on CRISPR/Cas9 gene therapy for cervical cancer revealed that CRISPR/Cas9 targeting E6 and E7 oncogenes of high-risk human papillomavirus 16 (HPV16) can significantly enhance the chemo-/radiosensitivity or prevent chemo-/radioresistance in chemo-/radiotherapy,^37,38^ and suppress in vitro and in vivo cell growth of cervical cancer cells, especially for the progression of tumorigenicity.^39^ In addition, suppression of triple-functional domain protein (Trio) using the CRISPR/Cas9 system was able to inhibit migration and invasion in cervical cancer.^40^ Our findings indicated that knockout of CD109 suppressed cell migration, growth, self-renewal and in vitro and in vivo tumorigenic capabilities in cervical cancer cell lines. CRISPR/Cas9 knockout of CD109 can abolish the tumorigenic and aggressive effects, which was highlighted in cervical squamous cell carcinoma.

However, the detailed mechanisms on how CD109 proteins promote tumour aggressiveness in cervical cancer are not clearly understood. CD109 has been identified as a co-receptor for TGF-β, which can bind to TGF-β1 to form a heteromeric complex with TGF-β receptors I and II (TGFβRI/TGFβRII), and enhances TGFβR1 proteasomal degradation.^13,41^ The addition of CD109 protein could lead to a downregulation of TGF-β signalling, and an upregulation of STAT3 phosphorylation, which might be further associated with the improvement of cell growth in human psoriatic keratinocytes.^17^ As it has been reported previously, CD109 interacted with EGFR in a glioblastoma cell line overexpressing CD109.^15^ Then, CD109 was assumed to potentially facilitate STAT3 signalling that would be mediated by EGFR dimerisation.^16^ In our study, we also investigated the effect of EGFR in the sorted CD109( + ) subpopulation with high expression of CD109 protein. Our data suggested that EGFR expression in CD109( + ) cells was higher than the CD109(−) cells. High expression of CD109 promoted EGFR-mediated STAT3 phosphorylation. Whilst blockade of CD109 by siRNA and CRISPR/Cas9 inhibited STAT3 activation in cervical cancer cell lines.

CD109 is known to play an important role in promoting tumorigenesis.^24–26^ A review^42^ pointed out that the crosstalk with EGFR signalling pathways would contribute to TGF-β effects. Furthermore, EGFR activation was associated with tumorigenic processes.^43^ In total, seven members of Signal Transducers and Activators of Transcription (STAT) family are known as STAT1, STAT2, STAT3, STAT4, STAT5A, STAT5B and STAT6, which could be activated by different extracellular cytokines, hormones and growth factors.^44,45^ STAT3 is regarded as an oncogene in the regulation of cell apoptosis, growth and carcinogenesis.^46,47^ In addition, STAT3 has been identified as a stem cell-specific regulator, and exhibits a female germline cell-specific binding pattern, which suggested that STAT3 might be strongly associated with the stemness-related regulation in cell signalling.^48,49^ Hence, EGFR and STAT3 might be the key effectors of the CD109-induced EGFR/STAT3 signalling network regulating the maintenance of CSC properties.

To examine whether CD109-induced EGFR-mediated STAT3 activation could contribute to the stemness-related regulation of CSCs, we performed anti-EGFR antibody treatment and siRNA STAT3 knockdown in sorted CD109( + ) CaSki cells, followed by in vitro functional assays. Firstly, we found that blockage of EGFR on the sorted CD109( + ) cells suppressed STAT3 activation at protein level, and remarkably reduced in vitro tumorigenicity and aggressiveness, as compared with the non-EGFR-blocked CD109-expressing cells. Then, we also found that STAT3 knockdown elicited similar cellular phenotypes as CD109 knockdown, remarkably reducing cell migration, viability and self-renewal ability. It has been reported that cancer cells expressing STAT3 and potential stem cell markers, Nanog and Oct-4, lost the plasticity to promote differentiation in human hepatocellular cancer, which means that together with Nanog and Oct-4, STAT3 would be essential for maintaining stem cell phenotype.^49^ The binding of phosphorylated STAT3 to the promoters of Bmi1 and Nanog gene would be promoted by chemotherapy drug, in which the STAT3 signalling partially mediates drug-induced stemness in pancreatic cancer cells.^50^ We determined the protein expression levels of several ‘stemness’ genes (Bmi1, Nanog and Oct-4A) on the sorted CaSki cells with/without STAT3 knockdown in this study. We found that enhanced protein expression level of these ‘stemness’ genes were detected in the sorted CD109( + ) subpopulation, whereas negative CD109 expression and knockdown of STAT3 attenuated the ‘stemness’ gene expression. STAT3 may have an essential role in maintaining the expression of those ‘stemness’ genes. To sum up, those findings suggested that CD109 induced EGFR-mediated STAT3 activation response for cell migrative and proliferative functions, and CSC phenotype maintenance in cervical cancer cell lines. It is hoped that further investigation of the functional effects on CD109/EGFR/STAT3 signalling may provide an insight for developing a cervical CSC-targeted therapeutic strategy.

In conclusion, our findings imply that CD109 mediates tumorigenicity and cancer aggressiveness in cervical squamous carcinoma cells. CD109 expression is frequently detected in cervical squamous cell carcinoma. Upregulation of CD109 protein enhances cell proliferation, migration, sphere-forming and anchorage-independent cell growth ability. In contrast, silencing of CD109 suppresses cell growth, migration and self-renewal capability. CRISPR KO of CD109 expression reverses the in vitro and in vivo tumorigenic and cancer-aggressive capability of cervical cancer cells. Moreover, CD109 is responsible for the induction of EGFR-mediated STAT3 regulation in cancer tumorigenicity and aggressiveness, which might reveal a potential molecular target for the development of an effective therapeutic strategy against cervical squamous cell carcinoma.

## Supplementary information

Supplementary Information

## Data Availability

The data sets used and/or analysed during the current study are available from the corresponding author on reasonable request.
