# Supplementary material for: CD109 mediates tumorigenicity and cancer aggressiveness via regulation of EGFR and STAT3 signalling in cervical squamous cell carcinoma
Source: Br J Cancer. 2020 Jun 8;123(5):833–43. doi: 10.1038/s41416-020-0922-7 (PMC7463003; doi:10.1038/s41416-020-0922-7)
Supplement: Supplementary file 1 — Supplementary Information [file 41416_2020_922_MOESM1_ESM.docx]

**Supplementary Table S1.** Correlation between clinicopathological factors and CD109 immunostaining.


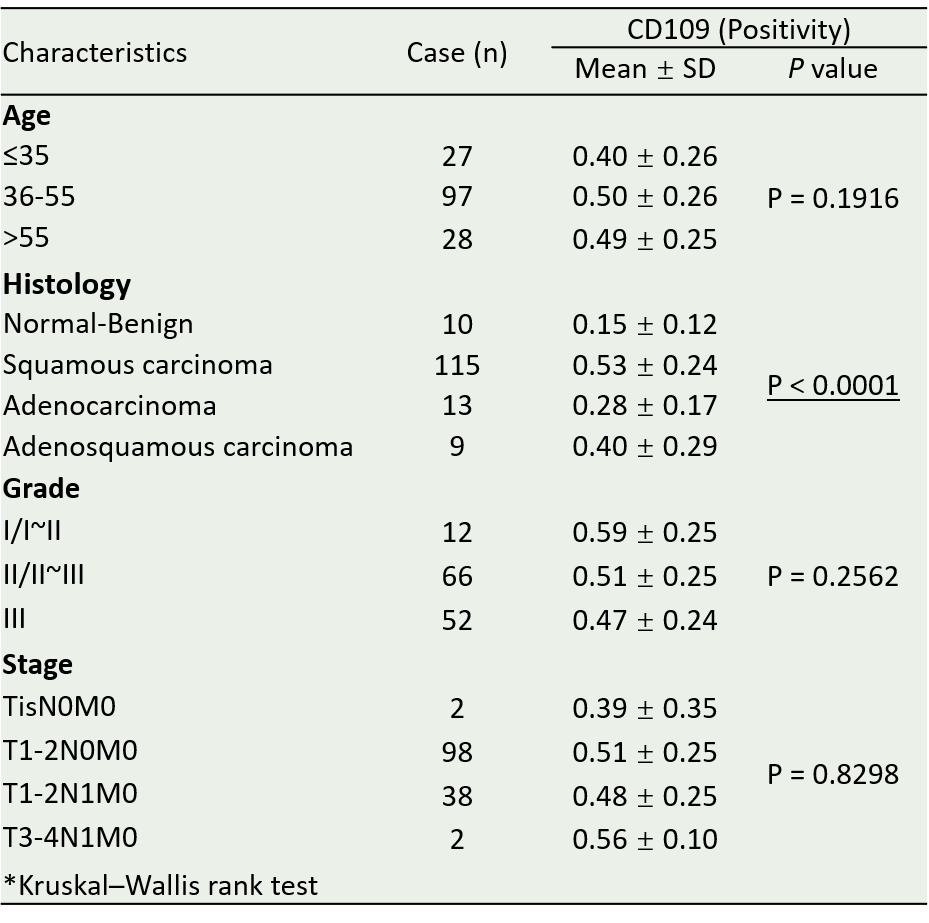


^*^ Kruskal–Wallis rank test was used to compare multiple subgroups of different clinical parameters. If there is a significant difference between multiple comparisons, then Dunn's multiple comparisons test and Mann-Whitney test would be applied for comparing data between two groups. These with significant P-values are underlined.


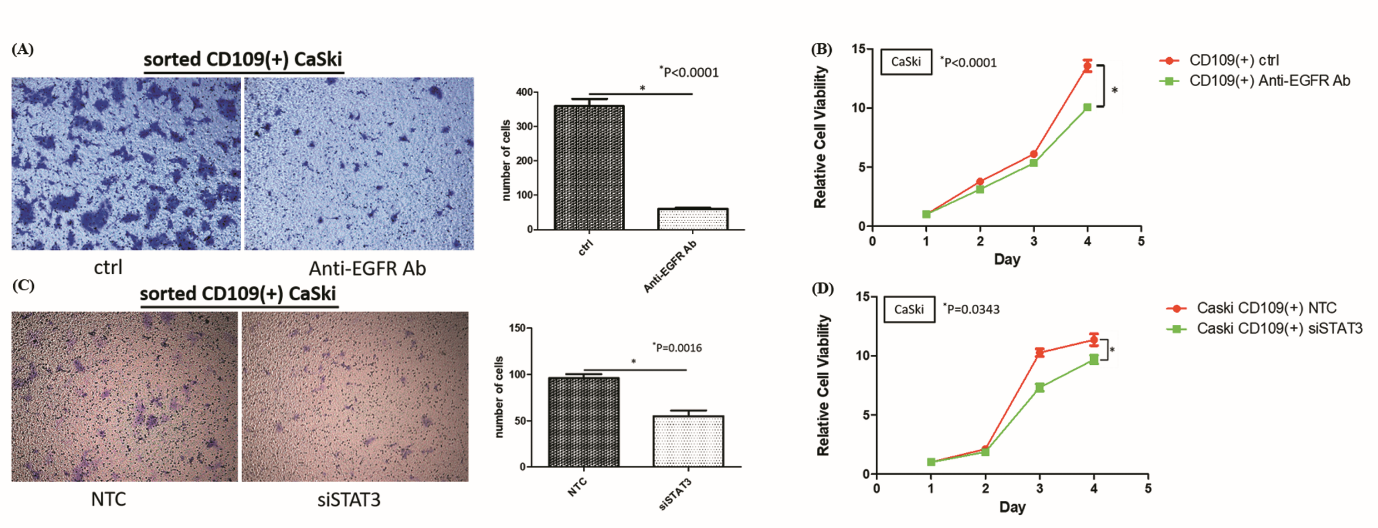
 **Supplementary Figure S1.** (A) A representative image (left) and quantification bar graph (right) of migrated CD109(+) CaSki cells treated w/o anti-EGFR antibody (crystal violet). ^*^ *P* < 0.0001. (B) Proliferation analyses of CD109(+) CaSki cells treated w/o anti-EGFR antibody. ^*^ *P* < 0.0001, using the Student’s t-test. (C) A representative image (left) and quantification bar graph (right) of migrated NTC siRNA or STAT3 siRNA knockdown CaSki CD109(+) cells (crystal violet). ^*^ *P* = 0.0016. (D) Proliferation analyses of NTC siRNA or STAT3 siRNA knockdown CaSki CD109(+) cells. ^*^ *P* = 0.0343.
